# Spontaneous nucleation and fast aggregate-dependent proliferation of α-synuclein aggregates within liquid condensates at neutral pH

**DOI:** 10.1073/pnas.2208792120

**Published:** 2023-02-21

**Authors:** Samuel T. Dada, Maarten C. Hardenberg, Zenon Toprakcioglu, Lena K. Mrugalla, Mariana P. Cali, Mollie O. McKeon, Ewa Klimont, Thomas C. T. Michaels, Tuomas P. J. Knowles, Michele Vendruscolo

**Affiliations:** ^a^Department of Chemistry, Centre for Misfolding Diseases, University of Cambridge, Cambridge CB2 1EW, UK; ^b^Department of Chemistry, Federal University of São Carlos, São Carlos, 13565-905 São Paulo, Brazil; ^c^Department of Biology, Institute of Biochemistry, ETH Zurich, 8093 Zurich, Switzerland; ^d^Bringing Materials to Life Initiative, ETH Zurich, 8093 Zurich, Switzerland

**Keywords:** phase separation, protein condensates, Parkinson’s disease

## Abstract

The aggregation of α-synuclein into amyloid fibrils has been under scrutiny in recent years because of its association with Parkinson’s disease. This process can be triggered by a lipid-dependent nucleation process, and the resulting aggregates can proliferate through secondary nucleation under acidic pH conditions. It has also been recently reported that the aggregation of α-synuclein may follow an alternative pathway, which takes place within dense liquid condensates formed through phase separation. The microscopic mechanism of this process, however, remains to be clarified. Here, we used fluorescence-based assays to enable a kinetic analysis of the microscopic steps underlying the aggregation process of α-synuclein within liquid condensates. Our analysis shows that at pH 7.4, this process starts with spontaneous primary nucleation followed by rapid aggregate-dependent proliferation. Our results thus reveal the microscopic mechanism of α-synuclein aggregation within condensates through the accurate quantification of the kinetic rate constants for the appearance and proliferation of α-synuclein aggregates at physiological pH.

## Significance

It has been recently shown that the speed of the conversion of α-synuclein from its native state into amyloid fibrils can be greatly enhanced by the formation of an intermediate dense liquid state formed through phase separation. The corresponding mechanism, however, remains to be fully characterized. In this work, we use a combination of fluorescence microscopy, microfluidics, and chemical kinetics to determine the microscopic rate constants for the primary nucleation and aggregate-dependent proliferation at neutral pH of α-synuclein fibrils. The approach that we report facilitates the study of the aggregation process of α-synuclein under physiological conditions and of its relationship with Parkinson’s disease and related synucleinopathies.

Parkinson’s disease is the most common neurodegenerative movement disorder (1, 2). A distinctive pathophysiological signature of this disease is the presence of abnormal intraneuronal protein deposits known as Lewy bodies (3, 4). One of the main components of Lewy bodies is α-synuclein (5), a peripheral membrane protein highly abundant at neuronal synapses (6, 7) and genetically linked with Parkinson’s disease (8, 9). This 140-residue disordered protein can be subdivided into three domains, an amphipathic N-terminal region (amino acids 1 to 60), a central hydrophobic region (non-amyloid-β component, or NAC, amino acids 61 to 95), and an acidic proline-rich C-terminal tail (amino acids 96 to 140) (7). Although α-synuclein aggregation is characteristic of Parkinson’s disease and related synucleinopathies, the corresponding mechanism and its possible pathological role in disease are not yet fully understood.

Generally, the aggregation process of proteins proceeds through a series of interconnected microscopic steps, including primary nucleation, elongation, and secondary nucleation (10, 11). During primary nucleation, the self-assembly of proteins from their native, monomeric form leads to the formation of oligomeric species, an event that may occur in solution or on surfaces including biological membranes (12, 13). The formation of these oligomers is typically a slow event governed by high kinetic barriers (10, 11). Once formed, the oligomers may convert into ordered assemblies rich in β structure, which are capable of further growth into fibrillar aggregates (14). In many cases, the surfaces of existing fibrillar aggregates then further catalyze the formation of new oligomers (15, 16). This secondary nucleation process is typically characterized by the assembly of protein monomers on the surface of fibrils that eventually nucleate into new oligomeric species (15, 16). This autocatalytic mechanism generates rapid fibril proliferation (15).

In the case of the aggregation process of α-synuclein, several key questions are still open, including two that we are addressing in this study. The first concerns whether there are cellular conditions under which α-synuclein can undergo spontaneous aggregation, and the second whether the proliferation of α-synuclein fibrils by aggregate-dependent feedback processes can take place at physiological pH. These questions are relevant because according to our current knowledge, α-synuclein aggregation does not readily take place spontaneously in the absence of contributing factors such as lipid membranes. Furthermore, secondary nucleation contributes significantly to the aggregation process only at acidic pH (13, 17). It thus remains challenging to rationalize the links between α-synuclein aggregation and Parkinson’s disease.

To address this problem, we investigated whether it is possible to leverage the recent finding that α-synuclein can undergo a phase separation process resulting in the formation of dense liquid condensates (18–21). Phase separation has recently emerged as a general phenomenon associated with a wide variety of cellular functions (22–25) and closely linked with human disease (23, 26–29). This process has been reported for a wide range of proteins implicated in neurodegenerative conditions, including tau, fused in sarcoma (FUS), and TAR DNA binding protein 43 (TDP-43) (30–32). Since it has also been shown that protein aggregation can take place within liquid condensates (19, 26, 32–36), we asked whether it is possible to characterize at the microscopic level the condensate-induced aggregation mechanism of α-synuclein by determining the kinetic rate constants of the corresponding microscopic processes.

To enable the accurate determination of the rate constants for the microscopic steps in α-synuclein aggregation within condensates, we developed fluorescence-based aggregation assays to monitor both the spontaneous aggregation of α-synuclein and the aggregation in the presence of aggregate seeds. Using these assays within the framework of a kinetic theory of protein aggregation (10, 11, 37), we show that α-synuclein can undergo spontaneous homogenous primary nucleation and fast aggregate-dependent proliferation within condensates at physiological pH.

## Results and Discussion

### Prerequisites for the Study of α-Synuclein Aggregation Following Phase Separation.

Increasing evidence indicates that aberrant protein aggregation can take place within dense liquid condensates generated through phase separation (18, 19, 21, 26, 32–35). Fluorescence recovery after photobleaching can be employed to monitor the progressive maturation of the liquid condensates (38), and the subsequent appearance of solid-like assemblies (18, 19) (*SI Appendix*, Fig. S1 and Movies S1 and S2). The accurate time-dependent measurement of the formation of ordered aggregate species, however, is still challenging. Therefore, the study of α-synuclein aggregation within condensates requires the development of a strategy to capture the initial stages of phase separation, the maturation of the resulting condensates, as well as the key microscopic steps that drive the subsequent conversion into amyloid aggregates.

### An In Vitro Strategy for the Study of α-Synuclein Aggregation within Liquid Condensates.

**Fig. 1.**
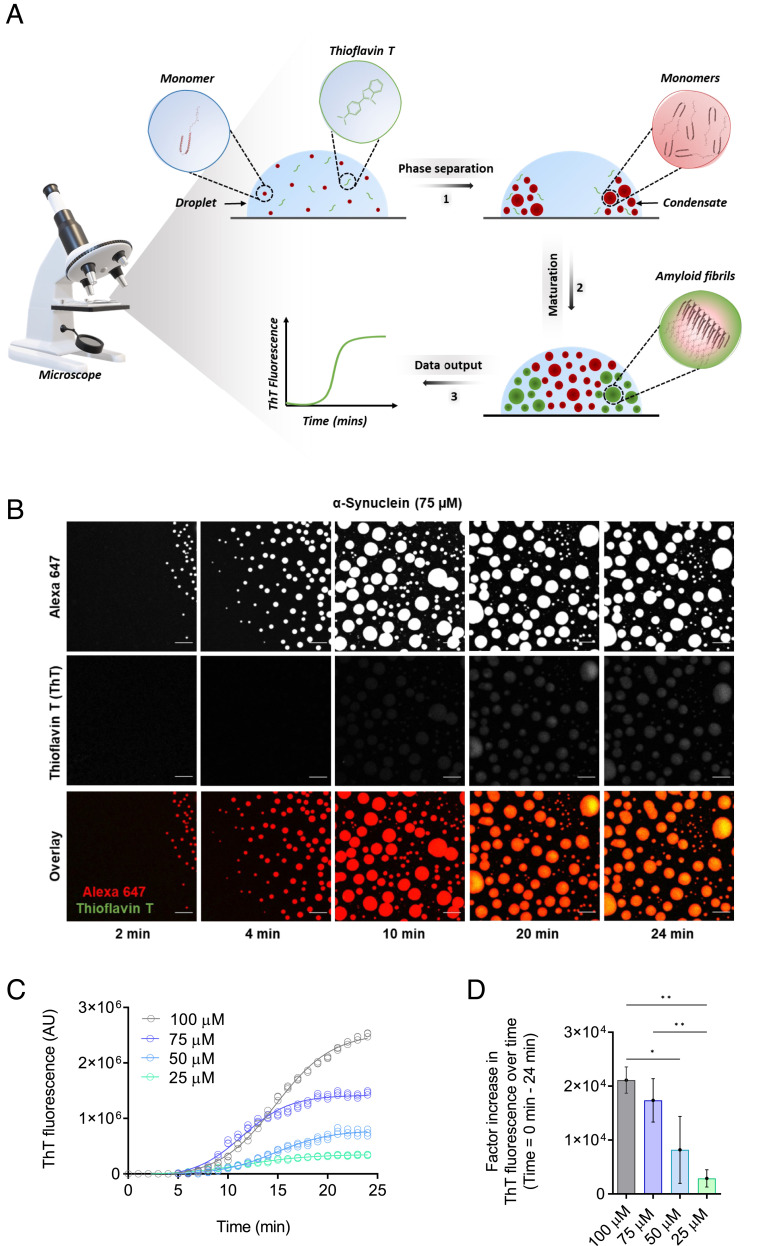
Development of a ThT-based assay to monitor α-synuclein aggregation within liquid condensates. (*A*) The assay has three components: 1) the phase separation of α-synuclein into dense liquid droplets (condensates) within dilute liquid droplets, which is monitored using Alexa Fluor 647 fluorescence, 2) the formation of α-synuclein amyloid fibrils over time within the condensates, and 3) the real-time assessment of amyloid formation is performed using ThT fluorescence. (*B*) Imaging of α-synuclein condensate formation (using Alexa Fluor 647 fluorescence) and aggregation (using ThT fluorescence) over time. In the presence of a crowding agent (10% PEG), α-synuclein monomers (75 μM) form a dense liquid phase surrounded by a dilute liquid phase. In the presence of 20 μM ThT, amyloid-containing condensates can be detected as an increase in the ThT fluorescence signal over time. The images represent an area of the sample that was tracked over time. (The scale bar represents 10 µm.) (*C*) Quantification of ThT fluorescence over time of the images shown in panel *B* for 75 μM (blue) α-synuclein. Subsequently, ThT emission for 100 (gray), 50 (cyan), and 25 (turquoise) μM α-synuclein were obtained in the same manner over 24 min. (*D*) Factor increase in ThT fluorescence intensities across the different concentrations tested (the ThT intensity at 24 min was subtracted by the ThT intensity at 0 min divided by the ThT intensity at 0 min). All experiments were performed in 50 mM Tris-HCl at pH 7.4 in the presence of 10% PEG and 20 μM ThT. The data represent the mean ± SEM of n = 3 individual experiments. One-way ANOVA., *P < 0.1, **P < 0.01.

To monitor closely the microscopic steps that drive α-synuclein aggregation within liquid condensates, we built on an established assay based on confocal microscopy used to observe rapid phase separation of α-synuclein in vitro (19). This assay enables the monitoring of α-synuclein condensates over time and is conducted by depositing a small sample volume as a droplet on a microscopy slide, where the phase separation process can be observed at physiological pH through the use of a molecular crowding agent, in our case polyethylene glycol (PEG) (Fig. 1 and *SI Appendix*, Fig. S2 *A*–*D*). Phase separation of α-synuclein can subsequently be characterized in real-time with fluorescence microscopy using a confocal microscope and α-synuclein labeled with Alexa Fluor 647. Next, to obtain a robust readout of the conversion of α-synuclein from its monomeric state to the amyloid state, we implemented the use of thioflavin T (ThT), a well-known benzothiazole fluorescent amyloid-binding dye employed for in vitro and in vivo characterization of amyloid formation (10, 19) (*SI Appendix*, Fig. S2*E*). ThT exhibits an enhanced fluorescent signal upon binding the cross-β structures characteristic of amyloid fibrils (10, 19). Our results indicate that ThT is soluble in PEG at the experimental conditions used in this work and can respond to changes in concentration of preformed α-synuclein fibrils (*SI Appendix*, Fig. S3).

We then aimed to investigate the ability of ThT to detect fibrillar species formed within α-synuclein condensates (Fig. 1 and Movie S3). To this end, monomeric α-synuclein labeled with the fluorophore Alexa Fluor 647 was incubated in the presence of 20 µM ThT and observed for about 20 min (Fig. 1*A*). This timeframe was chosen based on the observation that condensates transition from a liquid to a solid state about 20 min after the onset of phase separation (*SI Appendix*, Figs. S1 and S4). We observed a general increase in ThT fluorescence over time at various concentrations of α-synuclein, suggesting that fibrillar species had formed within the condensate (Fig. 1 *B*–*D*). During the first few minutes from the start of the phase separation process, the vast majority of α-synuclein was present in its monomeric state (Fig. 1 *B* and *C*). After 10 min, a notable increase in ThT emission was detected (Fig. 1 *B* and *C*), as evidenced by an exponential growth phase, which is indicative of a rapid increase in cross-β α-synuclein species within condensates (39). Such a speedy amplification of aggregate mass points toward the presence of secondary pathways, driving the amplification of cross-β aggregates (10, 11, 39). After approximately 18 min, the ThT emission reached a plateau, as most monomeric α-synuclein had been converted into fibrils (Fig. 1 *B* and *C*), indicating completion of the aggregation process.

To characterize their morphology at the end point of the assay, we performed transmission electron microscopy (TEM) imaging 24 min post the onset of phase separation. As expected, the TEM images indicated that α-synuclein fibrils had indeed formed. No fibrillar structures were observed prior to phase separation, and when individual components of the assay were assessed individually (*SI Appendix*, Fig. S5 *A* and *B*). Circular dichroism spectroscopy was employed to confirm the disordered, monomeric structure of α-synuclein prior to the observation of condensates. Fourier-transform infrared spectroscopy (FTIR) was then employed to characterize the structure of the amyloid aggregates formed pre and post phase separation (40, 41) (*SI Appendix*, Fig. S5 *C*–*E*). From the second derivative FTIR spectra, the prominent bands at 1,629 cm^−1^ and 1,627 cm^−1^ are indicative of β-sheet secondary structure composition (42) (*SI Appendix*, Fig. S5*E*). Taken together, the enhancement of ThT fluorescence, the observed fibrillar structures by TEM and prominent β-sheet infrared absorbance, which are all consistent with amyloid fibril formation, indicate that our assay is capable of monitoring the formation of α-synuclein fibrils following phase separation.

### The ThT-Based Aggregation Assay Is Sensitive to Changes in Phase Separation Behavior.

**Fig. 2.**
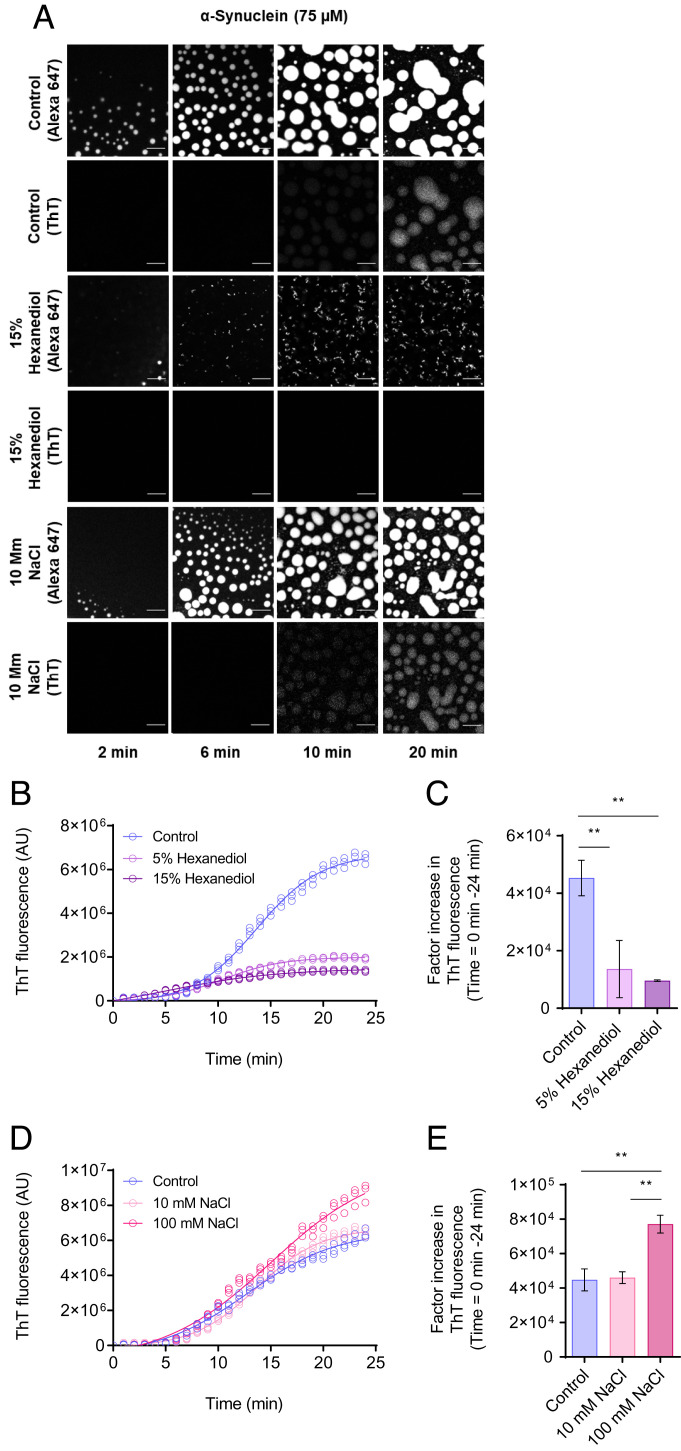
The ThT-based aggregation assay is sensitive to changes in the phase separation behavior. (*A*) Fluorescent images showing α-synuclein condensate formation (using Alexa Fluor 647 fluorescence) and aggregation (using ThT fluorescence) in the presence and absence (control) of 1,6-hexanediol (15% w/v), and 100 mM NaCl. The images suggest that the assay is sensitive to changes in the interactions between α-synuclein monomers as a result of 1,6-hexandiol and NaCl. (The scale bars represent 10 μm.) The images displayed represent an area of the droplet that was tracked over time. (*B*) Quantification of the ThT emission over time for the images shown in panel *A* for 75 μM α-synuclein (control) (blue) in the presence of 15% (w/v) (dark purple) and 5% (w/v) (light purple) 1,6-hexanediol over a 24-min period. (*C*) Factor increase in ThT fluorescence intensities from the data in panel *B* (the ThT intensity at 24 min was subtracted by the ThT intensity at 0 min divided by the ThT intensity at 0 min). (*D*) Rate of α-synuclein aggregation for the images in panel *A* in the presence and absence (blue) of 10 mM (light pink) and 100 mM (pink) NaCl over a 24-min period. (*E*) Factor increase in ThT fluorescence intensities from data in panel *D* (the ThT intensity at 24 min was subtracted by the ThT intensity at 0 min divided by the ThT intensity at 0 min). All experiments were performed using 75 µM α-synuclein in 50 mM Tris-HCl at pH 7.4 in the presence of 10% PEG and 20 µM ThT. The data represent mean ± SEM of n = 3 individual experiments. One-way ANOVA., *P < 0.1, **P < 0.01

As phase separation has been reported to depend on low-affinity electrostatic and hydrophobic interactions (43, 44), we used this feature to test the sensitivity of the aggregation assay described above in detecting changes in the protein interactions governing phase separation. A salt, sodium chloride (NaCl), and an aliphatic alcohol, 1,6-hexanediol, were chosen as controls due to their wide applications as effective tools to probe the material properties of protein condensates and inclusions (19, 45). NaCl alters the electrostatic interactions between protein molecules in the phase separation process, while 1,6-hexanediol works by interfering with the weak hydrophobic interactions that promote the formation of protein condensates. As expected, 1,6-hexanediol had a concentration-dependent inhibitory effect on the formation of α-synuclein condensates (Fig. 2 *A*–*C*). On the other hand, an increase in ionic strength led to a higher amyloid fibrillar yield (Fig. 2 *A*, *D*, and *E*), a result that implicates the involvement of the acidic C-terminal region of α-synuclein in its phase behavior. Taken together, these results demonstrate a reliable method of assessing α-synuclein aggregation within dense liquid condensates.

### A Framework to Elucidate the Mechanism of α-Synuclein Aggregation within Condensates.

Using the assay described above, we aimed to unravel the kinetic mechanisms by which monomeric α-synuclein transitions into fibrillar aggregates within liquid condensates. We first set out to establish a model of α-synuclein aggregation within condensates in an unperturbed system. Using the web-based software AmyloFit, we can characterize the complex reaction networks associated with protein aggregation using experimentally obtained kinetic data (39).

**Fig. 3.**
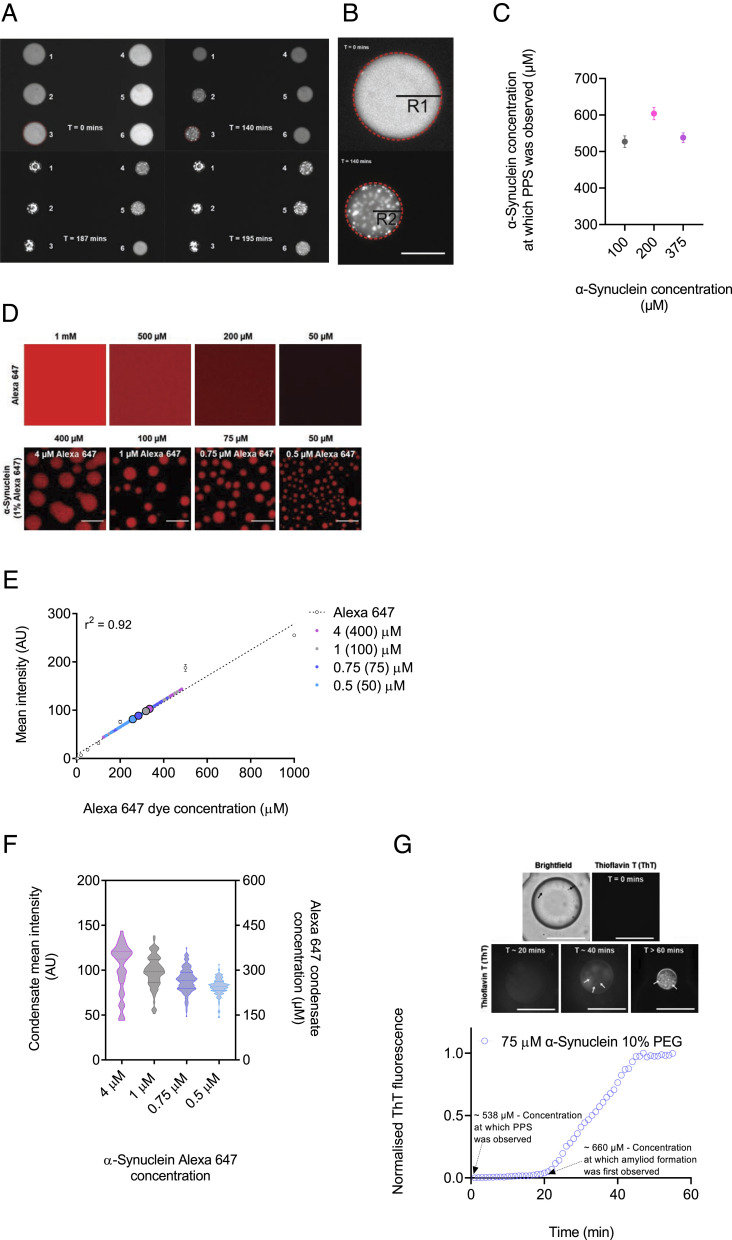
The aggregation kinetics of α-synuclein within condensates show a weak concentration dependence. Within condensates, normalized aggregation data at four α-synuclein concentrations (100, 75, 50, and 25 μM), yielded almost no variations between different concentrations (*SI Appendix*, Fig. S5 *A* and *B*). (*A*–*C*) The concentration required for phase separation of α-synuclein was obtained using a microfluidic device. (*A*) Representative fluorescence images displaying the timeline of condensate formation within several droplets (assigned a number from 1 to 6) trapped within a microfluidic chamber at 0, 140, 187, and 195 min (Movie S4). (*B*) Magnified image from panel *C* showing a droplet at the start (0 min) of the experiment and following initiation of phase separation (140 min). The concentration required for phase separation is obtained from the initial protein concentration and the change in droplet radius from the start (R1) of monitoring the droplet to the point at which phase separation is observed (R2). (The scale bar represents 50 µm.) (*C*) α-Synuclein concentration at which phase separation was observed within droplets as shown in panels *C* and *D*, at different initial protein concentrations (100 (gray), 200 (pink), and 100 (purple) μM) in the presence of 10% PEG, was calculated to be an average of 527, 604, and 538 μM, respectively (*SI Appendix*, Table S1). (*D*) Fluorescence images of Alexa Fluor 647 at a range of concentrations (1,000, 500, 200, and 50 μM) and images of monomeric α-synuclein (400, 100, 75, and 50 μM) condensates 10 min from the onset of phase separation. (The scale bar represents 10 µm.) (*E*) Fluorescence intensity of Alexa Fluor 647 from at a range of 1 to 1,000 μM from images shown in panel *D* was used to obtain a calibration curve of fluorescence signal for calibration (linear regression, r^2^ = 0.92). Small circles are the individual condensates measured for each α-synuclein concentration (n > 70 condensates per concentration), and big circles indicate the mean α-synuclein condensate intensity for each concentration [400 (light purple), 100 (gray), 75 (blue), and 50 (cyan) μM, and 4, 1, 0.75, and 0.5 μM for their respective 1% Alexa Fluor 647 protein concentration]. (*F*) Quantification of the mean fluorescence intensity (left Y axis) for individual condensates at each α-synuclein Alexa Fluor 647 concentrations 10 min from the onset of phase separation, and concentration within liquid droplets of α-synuclein labeled with Alexa Fluor 647 (right Y axis) at a range of bulk concentrations (400, 100, 75, and 50 μM) was estimated to be 335 ± 98, 319 ± 64, 284 ± 48 and 258 ± 34 μM after 10 min from the onset of phase separation (n > 70 condensates per concentration). (*G*) The concentration at which amyloid formation of α-synuclein was observed using a microfluidic device. Representative fluorescence images displaying the timeline of amyloid formation within droplet (75 μM α-synuclein, 10% PEG and 20 μM ThT) trapped within a microfluidic chamber at 0, 20, 40, and >60 min post protein phase separation (PPS). The α-synuclein concentration at which phase separation was observed within droplets was calculated to be an average of 538 ± 15.9 μM, and the concentration within droplet at which amyloid formation was observed was calculated to be an average of 660 ± 21.4 μM. (The scale bar represents 50 µm.) Data are shown for a representative experiment that was repeated at least three times.

Our data show that there is only a weak dependence of the aggregation kinetics on the total monomer concentration. This behavior is fundamentally different from that observed commonly for amyloid solution in dilute solution, where the nucleation and elongation steps are controlled by reaction rates that depend on the monomer concentration. By contrast, here, when the aggregation reaction takes place within a phase separated system, the local concentration within the condensed phase does not depend on the total protein concentration, the latter quantity only affecting the fraction of the system that is found in the condensed state (Fig. 3 and *SI Appendix*, Fig. S6 *A* and *B*).

This conclusion was further verified through monitoring the critical concentration for phase separation. To this effect, we used a microfluidic device, which trapped protein-containing aqueous microdroplets. In this way, we eliminated possible effects of the slide surface on the phase separation process in the confocal microscopy assay described above. By monitoring the microdroplets over time and by shrinking them in a controlled manner (*SI Appendix*, Fig. S6*C*), hence increasing the protein concentrations, we estimated the concentration of α-synuclein required for phase separation (Fig. 3 *A*–*C*). We observed that the concentration required for phase separation is independent of the initial protein concentration, but rather depends on the crowding agent (*SI Appendix*, Fig. S6 *D* and *E*). This observation could explain why a concentration-dependent effect cannot be observed in the kinetics, as we study the maturation of α-synuclein once phase separation is observed.

We then determined the monomeric protein concentration within the condensates as a function of time and of the monomeric protein concentration in the dilute phase. The fluorescence intensity of Alexa Fluor 647 was used as readout for monomeric protein concentrations, as this accounts for 1% (molar) of the total α-synuclein concentration (Fig. 3 *D*–*F*). We observed that the monomeric protein concentration within condensates, 6 min post phase separation did not change despite an increase in amyloid formation (*SI Appendix*, Fig. S6*F*). As condensates mature and grow in a concentration-dependent manner, we wanted to establish whether there was a correlation between the condensate size and the monomeric concentration of α-synuclein in the dense phase. At all tested concentrations, there was a weak correlation between these variables, and the differences of spread of data between concentrations were not significant (*SI Appendix*, Figs. S6*F* and S7). Given these results, we then estimated the concentration of labeled α-synuclein in the condensates to be around 300 to 400 μM. Since just 1% of α-synuclein was labeled, this result indicates that the total concentration of α-synuclein in the condensates is about 30 to 40 mM for all initial α-synuclein concentrations tested (400, 100, 75, and 50 μM). This estimate was done using a previously described method where the fluorescence intensity of individual α-synuclein condensates was measured, and Alexa Fluor 647 was used to make a calibration curve for fluorescence intensity (36) (Fig. 3 *D*–*F* and *SI Appendix*, Fig. S6*H*). Thus, the estimated total concentration of α-synuclein in condensates is about three orders of magnitude higher than in the diluted phase. The observation with respect to the lack of any significant difference in the variation in protein concentration within the condensates across all tested concentrations can be described by the phenomenon known as concentration buffering, which exist in phases separation of binary solutions, where variation in protein concentration in the bulk phase is buffered by the phase separated compartments (46, 47). Therefore, droplets with higher protein concentrations form larger condensates, whereas droplets with lower protein concentrations form smaller condensates (*SI Appendix*, Figs. S4*A* and S6*G*). Using the microfluids device described above, we also obtained the concentration at which amyloid formation was first observed in the droplets, which are shrinking during the experiments (Fig. 3*G*).

### Secondary Processes Dominate α-Synuclein Aggregation within Condensates.

**Fig. 4.**
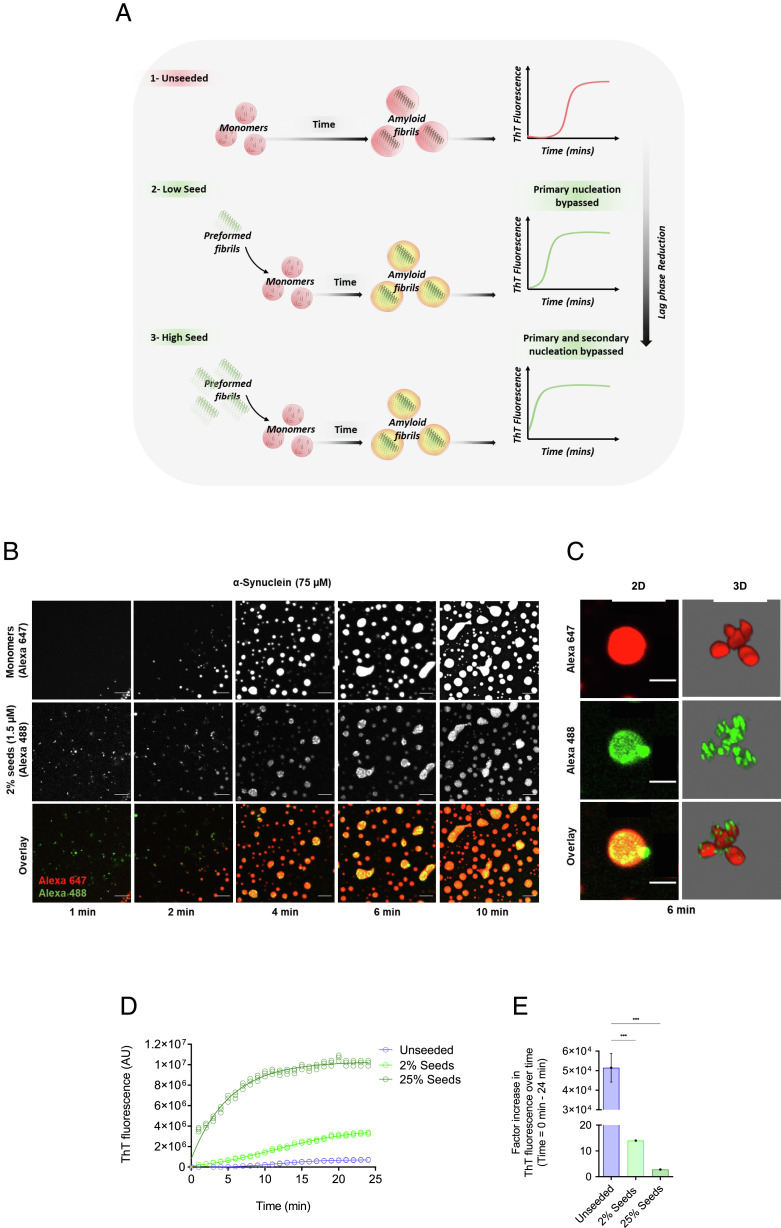
The addition preformed α-synuclein fibrils bypasses the primary nucleation of α-synuclein within condensates. (*A*) Schematic diagram illustrating the seeding process within the condensates. In the presence of preformed fibrils (seeds), aggregation is accelerated (2 and 3), whereas in the absence of seeds aggregation is slower (1). Increasing seed concentration results in a reduced lag time. (*B*) Representative fluorescence imaging displaying condensate formation by α-synuclein monomer (75 μM, labeled with Alexa Fluor 647) in the presence of 2% (1.5 μM) preformed α-synuclein fibrils (labeled with Alexa Fluor 488), 10% PEG in 50 mM Tris-HCl (pH 7.4). At 1 min, the presence of preformed fibrils within the droplet is observed in the dilute liquid phase. Upon the formation of droplets, preformed fibrils are seen to colocalize with condensates thereby accelerating aggregation by propelling the formation of aggregates. The images represent an area of the sample that was tracked over time. (The scale bar represents 10 µm.) (*C*) 2D and 3D rendered images of different droplets showing that preformed fibrils (labeled with Alexa Fluor 488) are directly recruited into α-synuclein condensates (labeled with Alexa Fluor 647) (Movie S5). (The scale bar represents 10 µm.) (*D*) Progression of ThT emission as a function of time (24 min) for 75 μM α-synuclein monomers (unseeded) (blue) with the addition of 2% (1.5 μM) (light green) and 25% (18.75 μM) (dark green) preformed fibrils. The data report on the increase in the ThT signal over time (unseeded, 2% seeds, 25% seeds). (*E*) Relative increase in the raw data values of the ThT fluorescence intensities from time from the onset of phase separation to 24 min post it. All aggregation assays were performed in 50 mM Tris-HCl (pH 7.4) in the presence of 10% PEG and 20 μM ThT. The results are shown as mean ± SEM. One-way ANOVA; ***P ≤ 0.001.

We next investigated the role of aggregate-dependent secondary processes on α-synuclein aggregation within condensates. The addition of preformed α-synuclein fibrils at the beginning of the aggregation assay is typically used as a qualitative way to check for the presence of secondary processes, as they act as seeds to aid in aggregate growth and multiplication (39). On these grounds, we aimed to bypass primary nucleation (with rate constant *k_n_*) events by adding a small amount of seeds (2%), where the rate of fibril formation is driven by secondary nucleation (with rate constant *k_2_*), fragmentation (with rate constant *k_−_*), and elongation (with rate constant *k_+_*) (15). The addition of large amounts (25%) of preformed seeds leads to primary and secondary nucleation being bypassed, and elongation of the preformed fibrils becoming the dominant process of aggregation (15) (Fig. 4*A*). If secondary processes are indeed present in α-synuclein aggregation within condensates, we expect a shortening in the lag phase in the presence of the preformed fibrils (Fig. 4*A*). Upon phase separation, preformed fibrils were observed to colocalize with the condensates, thus speeding up amyloid formation (Fig. 4 *B* and *C* and Movie S5).

Our results indicate that α-synuclein aggregation within condensates was accelerated in the presence of seeds as most newly formed fibrils were generated through secondary processes (Fig. 4*D*). In the presence of 25% preformed fibrils, α-synuclein aggregation was accelerated drastically to the extent that the plateau phase was reached 10 min post phase separation (Fig. 4 *D* and *E*). Visually, the presence of preformed fibrils within our assay was observed prior to phase separation (Fig. 4*B*). Overall, the data suggest that secondary processes are involved in the aggregation of α-synuclein within condensates. To confirm this result, we calculated the concentration of preformed fibrils within the condensates.

**Fig. 5.**
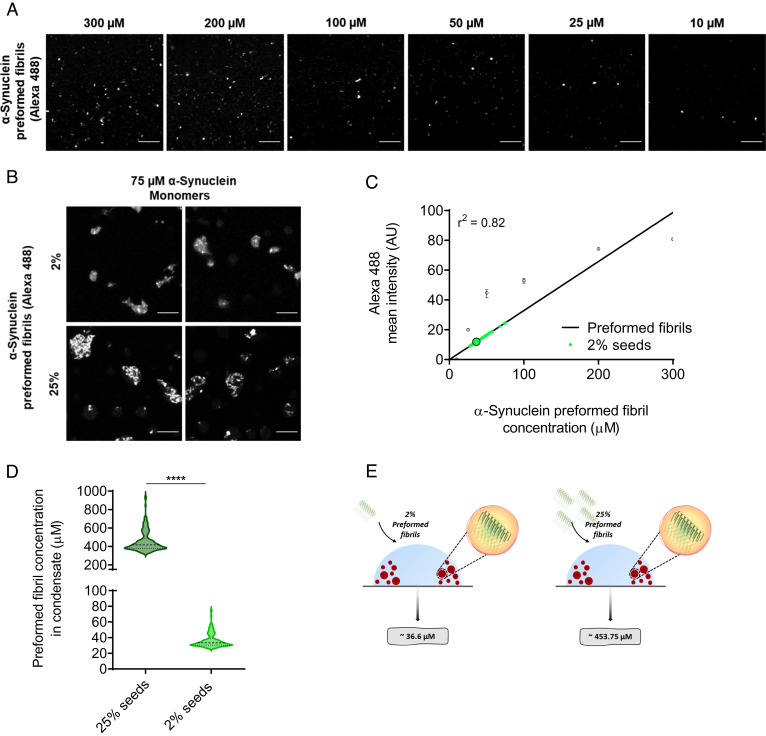
Determination of the concentration of preformed α-synuclein fibrils within α-synuclein condensates. (*A*) Fluorescence images of preformed α-synuclein fibrils (labeled with Alexa Fluor 488) at decreasing concentrations (300, 200, 100, 50, 25, and 10 μM). (The scale bar represents 10 µm.) (*B*) Representative fluorescence imaging displaying colocalization of 2% and 25% preformed fibrils (labeled with Alexa Fluor 488) within α-synuclein condensates (labeled with Alexa Fluor 647) 10 min post phase separation. (The scale bar represents 10 µm.) (*C*) The data reported in panel *A* were used for the calibration of the Alexa Fluor 488 fluorescence signal (linear regression, r^2^ = 0.82). The small circles are the individual condensates (n > 100) measured 10 min from the onset of phase separation and the big circle indicates the mean intensity of all fluorescently labeled preformed α-synuclein fibril within condensates. The Alexa Fluor 488 fluorescence signal of condensates in the assay containing 25% preformed fibrils had a higher intensity than the intensity of 300 μM preformed fibrils used for the calibration curve. (*D*) The average concentration of 2% (1.5 μM) and 25% (18.75 μM) preformed α-synuclein fibrils (labeled with Alexa Fluor 488) within α-synuclein condensates (75 μM) was estimated as 36 μM (SD = ±9, Min = 27, Max = 75 μM) and 454 μM (SD = ±112, Min = 341, Max = 939 μM), respectively. The results refer to 10 min from the onset of phase separation (n > 100 condensates). (*E*) Schematic illustrating the total concentration of preformed α-synuclein fibrils within condensates to be approximately 36.3 and 453.75 µM for 2% and 25% seeds, respectively. Data are from a representative experiment repeated three times with similar results. All seeded aggregation experiments were performed in 50 mM Tris-HCl (pH 7.4) and 10% PEG. The results are shown as mean ± SEM. One-way ANOVA; ****P ≤ 0.0001.

To estimate the concentration of preformed α-synuclein fibrils within condensates, several condensates containing fluorescently labeled preformed fibrils were assessed for their fibril content from several time points between 6 and 20 min (Fig. 5). We estimated the concentration of preformed α-synuclein fibrils within condensates to be 37 μM for 2% seeds (1.5 μM). We obtained this result by monitoring the intensity of individual α-synuclein condensates (formed from an initial concentration of 75 μM monomeric α-synuclein) containing preformed fibrils, and using a range of Alexa Fluor 488 fibril concentrations (50 to 300 μM) to create a calibration curve of Alexa Fluor 488 mean fluorescence intensity (Fig. 5 *A*–*D*). With regard to 25% seeds (18.75 μM), the average mean fluorescence intensity was greater than that of the highest concentration (300 μM) used for the calibration curve (Fig. 5*C*). In view of this, the equation $c^{I}≅24{.2c}^{\mathrm{tot}}$ was derived to estimate the concentration of α-synuclein in fibrils (referred to as fibril concentration) inside the condensate (*Methods*), where 24.2 is the constant obtained from the slope of the calibration curve, $c^{I}$ refers to the fibrils partitioned inside the condensate, and $c^{\mathrm{tot}}$ refers to the total fibril concentration added to the system (1.5 and 18.75 μM) (Fig. 5*C*). Using Equations 1 to 5 (*Methods*), the concentration of preformed fibrils within condensates at 25% seeds was estimated to be at 454 μM (Fig. 5 *A*–*D*).

**Fig. 6.**
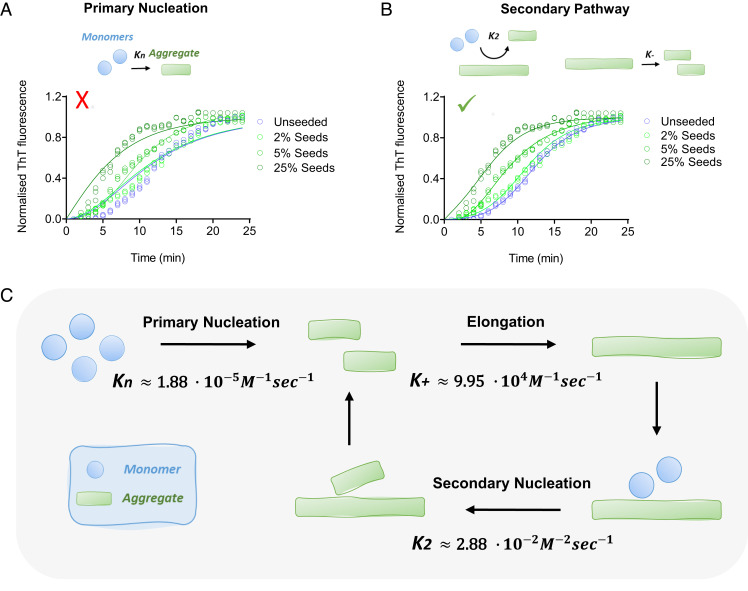
Secondary processes dominate α-synuclein aggregate proliferation within liquid condensates at physiological pH. (*A* and *B*) Analysis of the results of the ThT aggregation assay using AmyloFit, with the normalized aggregation curves measured as a function of time from the unperturbed system (blue), as well as in the presence of 2% (light green), 5% (green) and 25% (dark green) seeds. The graphs represent the best fits of different models in which, respectively, primary nucleation (*A*) and secondary processes (*B*) are assumed to be the dominant mechanism of aggregation. The mean residual errors were 0.009 (with $n_{c}=2)$ (*A*) and 0.002 with ( $n_{c}=2,$ $n_{2}=1$ ) (*B*). The solid lines represent the best fit to each respective condition. We used $m_{0}=30\;mM$ in all fits. For the unseeded assay we used $M_{0}=0$ , and for the seeded assay we used $M_{0}=36.3$ , 90.75 and 453.75 μM for 2%, 5% and 25% seeds, respectively. All experiments were performed three times using 75 µM α-synuclein in 50 mM Tris-HCl (pH 7.4) in the presence of 10% PEG and 20 µM ThT. (*C*) Illustration of the microscopic processes involved in α-synuclein aggregation, in the case when fragmentation is not contributing significantly, using the assay reported in this work. The associated rate constants are also reported. Despite the low values of the rate constants for primary nucleation and secondary nucleation, the aggregation process proceeds rapidly within the dense phase because of the high concentration of α-synuclein.

Having obtained the concentrations of preformed fibrils within condensates, we tested the fit of the seeded kinetics data to two different models (Fig. 6). The first fit shows that the aggregation data are not well described by a model that includes only primary nucleation and elongation. In contrast, the model with secondary processes resulted in a good fit (Fig. 6). As primary nucleation and secondary nucleation have a different concentration dependence, if we assume that fragmentation does not contribute significantly to aggregate proliferation under the conditions used (no shaking), the critical fibril concentration at which secondary nucleation becomes the dominant mechanism is 20 mM in our system (ratio of the primary to secondary nucleation rate constants (15), $K_{n}/K_{2}m_{o}$ , Fig. 6).

## Conclusions

We have determined the microscopic processes governing the aggregation of α-synuclein within liquid condensates (Fig. 6). Our approach has enabled the quantification of the rate constants for α-synuclein spontaneous homogenous primary nucleation and rapid aggregate-dependent aggregate proliferation at physiological pH. We anticipate that this approach will promote further studies to establish whether α-synuclein aggregation via the phase separation pathway is relevant to the onset and progression of Parkinson’s disease. In perspective, the possibility of studying the aggregation process of α-synuclein under physiological conditions with the fast and reliable assays that we have reported may open opportunities for screening compounds to inhibit α-synuclein aggregation and its pathological consequences in Parkinson’s disease and related synucleinopathies.

## Materials and Methods

The methods of purification and labeling of the proteins are described in *SI Appendix, Materials and Methods*. The methods of monitoring the phase separation and the aggregation of the proteins are also described in *SI Appendix, Materials and Methods*. The details of all experimental assays are reported in *SI Appendix, Materials and Methods*.

## Supplementary Material

Appendix 01 (PDF)

Movie S1. Formation of α-synuclein condensates (75 μM) at the edge of a drop in 50 mM Tris, pH 7.4 and 10% PEG; the scale bar represents 20 μm.

Movie S2. Fusion events of several α-synuclein condensates after 7 min post the onset of phase separation; the scale bar represents 20 μm.

Movie S3. α-Synuclein condensates formation and maturation, and α-synuclein aggregation at near-physiological concentration (75 μM) and conditions (50 mM Tris, pH 7.4) in the presence of 20 μM ThT; Alexa 647 channel (red) ThT channel (green) and merged channels; the scale bar represents 10 μm.

Movie S4. Time-lapse of formation of α-synuclein condensates in droplets trapped within the microfluidic device.

Movie S5. 3D rendered composition from Z-stacked images showing preformed fibrils (Alexa Fluor 488 - green) co-localised with α-synuclein condensates (Alexa Fluor 647 - red).

## Data Availability

All study data are included in the article and/or supporting information.
