# Supplementary material for: Spontaneous nucleation and fast aggregate-dependent proliferation of α-synuclein aggregates within liquid condensates at neutral pH
Source: Proc Natl Acad Sci U S A. 2023 Feb 21;120(9):e2208792120. doi: 10.1073/pnas.2208792120 (PMC9992821; doi:10.1073/pnas.2208792120)
Supplement: Supplementary file 1 — Appendix 01 (PDF) [file pnas.2208792120.sapp.pdf]

Supplementary Information

Purification and labelling of α-synuclein

Human wild-type α-synuclein and its cysteine variant A90C were expressed and purified as previously described, using *E. coli* BL21 (DE3)-gold competent cells (Agilent Technologies) transformed with the pT7-7 plasmid encoding α-synuclein. Expression was induced by the addition of 1 mM isopropyl β-d-1-thiogalactopyranoside (IPTG) (1). Following purification, the proteins were concentrated, and buffer exchanged into either 50 mM trisaminomethane-hydrochloride (Tris-HCl), 20 mM sodium phosphate (NaPi) or phosphate buffered saline (PBS) at pH 7.4 using Amicon Ultra-15 Centrifugal Filter Units (Merck Millipore). The A90C variant was labelled with 1.5-fold molar excess of C5 maleimide-linked Alexa Fluor 647 (Invitrogen Life Technologies) overnight at 4 °C under constant gentle stirring. The unbound dye was removed using Amicon Ultra-15 Centrifugal Filter Units and buffer exchanged into either 50 mM Tris-HCl, 20 mM NaPi or PBS at pH 7.4. The final protein concentration was measured using ultraviolet-visible (UV-vis) spectroscopy on a Cary 100 system (Agilent Technologies). All proteins were aliquoted, flash-frozen in liquid nitrogen, stored at -80 °C and thawed once before each experiment.

Preparation and labelling of preformed α-synuclein fibrils

Preformed α-synuclein fibrils were formed from recombinant α-synuclein wild-type or cysteine variant (A90C) monomers diluted in buffer (50 mM Tris-HCl at pH 7.4) to concentrations of approximately 500 μM. Monomers in microcentrifuge Eppendorf tubes (Axygen low-bind tubes) were incubated at 40 °C, with constant stirring speed (1,500 rpm) with a teflon bar and left to fibrillate on an RCT Basic Heat Plate (RCT Basic, model no. 0003810002; IKA, Staufen, Germany) for up to 72 h. Samples were centrifuged (Centrifuge 5427 R) at 4 °C, 14,000 rpm for 15 min. The supernatant was used to determine the monomer concentration and fibril concentration was determined as monomer equivalents. For visualisation purposes, A90C α-synuclein fibrils were labelled with 1.5-fold molar excess of C5 maleimide-linked Alexa Fluor 488 (Invitrogen Life Technologies) overnight at 4 °C under constant but gentle stirring. The excess dye was removed by at least 3 sequential 50 mM Tris-HCl washes by centrifugation (4 °C, pH 7.4, 14,000 rpm) for 15 min. Before each experiment, seed fibrils were pre-treated by 15 sec (15 pulses) sonication at 10% power with 50% duty cycle using a Microtip sonicator (Bandelin Sonopuls HD2070) to disperse lumped fibrils.

α-Synuclein phase separation assay

Non-labelled wild-type α-synuclein was mixed with the A90C variant labelled with Alexa Fluor 647 at a 100:1 molar ratio in either 50 mM Tris-HCl, 20 mM NaPi or PBS (pH 7.4) and 10% polyethylene

glycol 10,000 (PEG) (Thermo Fisher Scientific) or labelled maleimide PEG FITC (Fluorescein) 10,000 (NANOCS) by volume at room temperature (20-22 °C). Additionally, NaCl, 1,6-hexanediol and dimethyl sulfoxide (DMSO) and preformed fibrils were supplemented in the phase separation assay. 10 μL of the final mixture was pipetted on a 35 mm glass bottom dish (P35G-1.5-20-C, MatTek Life Sciences) and immediately imaged on a Leica TCS SP8 inverted confocal microscope using a 60×/1.4 HC PL Apo CS oil objective (Leica Microsystems). The excitation wavelength was set to 633 nm for all experiments. All images were processed and analysed in ImageJ (NIH).

Fluorescence spectra

The excitation and emission spectra of 20 mM solutions of thioflavin T (ThT), Alexa Fluor 488 and Alexa Fluor 647 were obtained on a Cary Eclipse fluorescence spectrometer (Agilent). The emission and excitation wavelength used were λ_{ex}=440 nm/λ_{em}=480 nm for ThT, λ_{ex}=496 nm/λ_{em}=520 nm for Alexa488 and λ_{ex}=650 nm/λ_{em}=665 nm for Alexa647. The spectra were plotted using Graphpad Prism.

Preformed fibril bound thioflavin T (ThT) assay

To study the concentration-dependent emission of fibril-bound ThT, wild-type α-synuclein preformed fibrils (100, 75, 50, 20 and 10 μM) were incubated with 20 μM ThT in 50 mM Tris-HCl and 10% PEG. For control monomeric wild-type α-synuclein were also treated with 20 μM ThT in 50 mM Tris-HCl and 10% PEG. 10 μL of the final mixture was pipetted on a 35 mm glass bottom dish and immediately imaged on a Leica TCS SP8 inverted confocal microscope using a 60×/1.4 HC PL Apo CS oil objective (Leica Microsystems). The excitation wavelength was 488 nm for all ThT-based experiments. All images were processed and analysed in ImageJ (NIH).

Fluorescence recovery after photobleaching (FRAP) experiment

FRAP experiments were performed on condensates using a Leica Stellaris Will inverted stage scanning confocal microscope. To conduct FRAP experiments, a 63x magnification oil objective (63x/1.4 HC PL Apo CS oil) was used. Bleaching was done using the 647 nM laser at 20% intensity for 2 sec following a 2 sec pre-beach sequence. Immediate post-beach images were captured at 500 ms per frame rate for 55 sec. Intensity traces of bleached area were background corrected and normalised to reference signal and FRAP time which is defined by the time to half maximal signal recovery. This was determined using the FRAP wizard system (Leica) on the confocal microscope.

Transmission electron microscopy (TEM)

α-Synuclein samples from the ThT-based aggregation assay were obtained prior to fibril formation mediated by phase separation and after fibril formation. The obtained sample (5 μL) was deposited on a carbon film of 400 mesh 3 mm copper grid. The grids were washed once with Milli-Q water, then

incubated with 1% (w/v) uranyl acetate for 2 min and washed twice again with Milli-Q water before being air-dried at room temperature. Samples were imaged at the Cambridge Advanced Imaging Centre using a Tecnai G2 transmission electron microscope operating at 80–200 keV.

Circular dichroism (CD)

Protein samples spectra containing non-labelled wild-type α-synuclein was mixed with the A90C variant labelled with Alexa Fluor 647 at a 100:1 molar ratio in 50 mM Tris-HCl, (pH 7.4), 10% polyethylene glycol 10,000 (PEG) were diluted 1:10 in 50mM TRIS (pH 7.4) and 20 μM ThT. were obtained on a JASCXO J-810 (Easton, USA) using quartz cuvettes with path lengths of 1mm by averaging 8 separate spectra each recorded between 200nm-250nm, with 1nm bandwidth, 0.5nm data pitch, scanning speed of 50nm/min and a 1s response time.

Fourier-transform infrared spectroscopy (FTIR)

Product from phase separation assay prior to condensate formation and upon amyloid formation were washed off glass slide with 50 mM Tris-HCL, 50 μM monomeric α-synuclein were seeded with 1 μL of product formed through phase separation at unknown concentration. Reaction mixture left in a plate reader for 24 h to aggregate quiescently. Once ThT fluorescence reached saturated, reactions were stopped, and products were recovered from microplate wells. Product was centrifuged at 12,000 rpm for 10 min, at 4 °C. Supernatant containing soluble α-synuclein was discarded, and pellets were washed twice with H_2O. Pellets were resuspended in 3 μL H_2O and subjected to attenuated total reflectance (ATR) Fourier transform infrared (FTIR) spectroscopy. 1.5 μL of solute was applied to a Perkin-Elmer Spectrum100 FTIR with an ATR diamond attachment and dried using dry air. Prior to scanning, sample and electronics chambers were purged with a constant flow of dry air. 100 replicate scans were averaged from 4000 to 800 cm^{-1}, normalized to amide I intensity (~ 1630 cm^{-1} peak), and second derivatives were taken with 9 points for slope analysis.

α-Synuclein aggregation assay within dense liquid condensates

20 or 40 μM ThT (Sigma) and additional compounds and preformed fibrils, depending on the experiments, were mixed with monomeric wild-type α-synuclein, containing 1 molar % A90C α-synuclein labelled with Alexa Fluor 647 prior to each experiment. The assay mixture, which included 50 mM Tris-HCl, pH 7.4, 10% PEG 10,000, was pipetted onto a 35 mm glass bottom dish and imaged on a Leica TCS SP8 inverted confocal microscope using a 60×/1.4 HC PL Apo CS oil objective. The excitation wavelength 633 nm and 488 nm were used for Alexa Fluor 647 labelled α-synuclein and ThT, respectively. Images were processed in ImageJ; an area adjacent to the edge of the droplet was cropped and analysed, thus allowing for time-dependent measurement of amyloid formation.

Estimation of the α-synuclein concentration required for phase separation in water-in-oil droplets

Fabrication of microfluidic devices. The fabrication process of the microfluidics was taken from a previously established protocol (2-4). In brief, a soft photolithographic process was used to fabricate the master through which microfluidic devices were made. A 50 µm photoresist (SU-8 3050, MicroChem) was spin-coated onto a silicon wafer. This was soft baked at 95 °C for 3 min. A film mask was placed on the wafer and the whole system was exposed in UV light to induce polymerization. The wafer was then baked at 95 °C for 30 min. Finally, the master was placed into a solution of propylene glycol methyl ether acetate (PGMEA, Sigma-Aldrich), which helped in the development process. Elastomer polydimethylsiloxane (PDMS) with curing agent (Sylgard 184, DowCorning, Midland, MI) was mixed at a ratio of 10:1 to fabricate the devices. This mixture was then incubated at 65 °C and cured for a total of 3 h. Once hardened, the PDMS was peeled off the master, and holes were punched into the PDMS, which acted as inlets and outlets. Finally, the PDMS slab was bound to a glass slide by treatment with a plasma bonder (Diener Electronic, Ebhausen, Germany).

Formation and confinement of droplets. neMESYS syringe pumps. Syringe pumps (Cetoni, Korbussen, Germany) were used to control the flow rates within the microchannels. Protein solution was mixed with PEG at a ratio of 1:1 at the first junction. At the second junction, the oil phase, which consisted of fluorinated oil (Fluorinert FC-40, Sigma-Aldrich) and 2% w/w fluorosurfactant (RAN biotechnologies) intersected the aqueous phase resulting in water-in-oil droplets being formed. Following droplet generation, droplets were confined within a microfluidic trap (3, 4). In brief, droplets are directed towards an array of traps whereby once a droplet is driven within the microfluidic confinement, it is unable to escape unless a pressure is applied from the outlet. Droplets were then incubated at room temperature to allow for shrinkage. This resulted in an increase of the local concentration of protein and PEG within the droplets which led to protein phase separation. The water-in-oil droplets and the protein phase separation was monitored using fluorescence microscopy.

Calculation of protein concentration during phase separation. The concentration of the protein was obtained by calculating the ratio of the droplet volume just after trapping (V_1) and at the point of phase separation (V_2), i.e. at the point at which condensates start appearing within the water-in-oil droplet. By multiplying the initial monomeric protein concertation by the value of this ratio, the actual protein concentration at the point of phase separation could be determined.

Estimation of α-synuclein concentration in condensates

The fluorescence calibration was performed by imaging solutions of the Alexa Fluor 647 dye at 1000, 500, 200, 100, 50, 20 and 1 mM in 50 mM Tris-HCl at pH 7.4 with the addition of 10% PEG. Using the same acquisition settings, images were taken of α-synuclein condensates at 400, 100, 75, 50 and 25 µM 10 min after phase separation to estimate the local concentration of α-synuclein in condensates.

Several condensates (approximately 70 per concentration) were analysed by drawing a circular region of interest around the condensate to obtain the average pixel intensity. Linear regression analysis of the intensity of Alexa Fluor 647 calibration images (r^2 = 0.92) was used to interpolate the estimated concentration of α-synuclein proteins within condensates.

Estimation of the concentration of preformed α-synuclein fibrils in condensates

A calibration of the Alexa Fluor 488 fluorescence intensity was carried out using various fibril concentrations of A90C α-synuclein labelled with Alexa Fluor 488 in 50 mM Tris-HCl at pH 7.4 with the addition of 10% PEG. Using the same acquisition settings, images were taken of α-synuclein condensates 6 min after phase separation at 75 µM containing 2% and 25% Alexa Fluor 488 labelled preformed fibrils to estimate the local concentration of preformed fibrils within the liquid droplet. Several condensates (approximately 100) were analysed by drawing a circular region of interest around the condensate to obtain the average pixel intensity for each droplet. Linear regression analysis of the intensity of Alexa Fluor 488 fibrils images (r^2 = 0.82) was used to estimate the concentration of α-synuclein preformed fibrils within condensates (**Figure 4C**).

If we consider a system of total volume V and containing a dense phase coexisting with a dilute phase, the dense phase (which we term phase I) occupies a volume V^I, while the volume of the dilute phase (which we term phase II) is $V^{II} = V - V^I$, such that

$$V = V^I + V^{II} \tag{1}$$

If we add to this system a total concentration c^{tot} of preformed fibrils, these fibrils will partition differently in phases I and II depending on their relative interaction with the dense and dilute phases. We capture this behaviour by means of the partitioning coefficient Γ, defined as

$$\Gamma = \frac{c^I}{c^{II}} \tag{2}$$

While Γ can be determined from the Flory-Huggins theory of phase separation (5), we will consider it as a known parameter in our calculations, where c^I and c^{II} are the concentrations of preformed fibrils in phases I and II, respectively. The total fibril concentration in the system is the average of the concentrations c^I and c^{II} weighted by the volumes of the respective phases

$$c^{tot} = \frac{V^I}{V} c^I + \frac{V^{II}}{V} c^{II} \tag{3}$$

This condition states that the total number of fibrils in the system is conserved. By combining Eqs. (1), (2) and (3) we can solve for c^I and c^{II} and find

$$c^I = \xi\, \Gamma\, c^{tot}, \qquad c^{II} = \xi\, c^{tot} \tag{4a}$$

where

$$\xi = \frac{1}{1+(\Gamma-1)\, V^I/V} \tag{4b}$$

Using Eq. (4) we can calculate the concentration of fibrils in the condensed phase from c^{tot}, Γ and V^I/V. In the limit when $\Gamma \rightarrow \infty$ (when the fibrils partition mostly inside the condensed phase), Eq. (4) reduces to

$$c^I = \frac{V}{V^I} c^{tot}, \qquad c^{II} = 0 \qquad (5)$$

In this regime, we have a linear relationship linking c^I to c^{tot}. The slope of this relationship is V/V^I and relates to the volume fraction V^I/V occupied by the dense phase. In our experiments we measured $c^I = 36.6$ µM for $c^{tot} = 1.5$ µM and $c^I = 453.75$ µM for $c^{tot} = 18.75$ µM. With these data points we estimated a slope $V/V^I = 24.2$.

Image analysis of α-synuclein condensates

Images of wild-type α-synuclein, containing 1 molar % α-synuclein Alexa Fluor 647 labelled A90C α-synuclein were acquired on a Leica TCS SP8 inverted confocal microscope system. All images within an experiment were acquired using identical confocal settings (scan speed, resolution, magnification, laser intensity, gain, and offset). Images were analysed by applying threshold functions in ImageJ software that identified the phase separated α-synuclein condensates and excluded the background of the image. All condensates within the threshold limits were analysed for total area (µm^2), average size of individual droplets (µm^2) and mean fluorescence intensity of individual condensates (arbitrary units) (**Figure S6**).

AmyloFit data analysis

The experimental ThT fluorescence readout was uploaded on the free online platform AmyloFit(6) (https://www.amylofit.ch.cam.ac.uk). The software normalised the data to 0% and 100% by averaging the values at the baseline and the plateau of the reaction. Upon data normalisation, the concentration of aggregate mass could be observed as a function of time. A basin-hopping algorithm was applied to fit the experimental data to a model of protein aggregation. The initial monomer concentration (m_0) was

set to 30 mM. Reaction orders for primary and secondary nucleation were set to 2. The number of basin hops was set to 40 for each fit. The AmyloFit user manual can be consulted for more in-depth information on the fitting procedure (6). We note that at the high concentrations of α-synuclein in the condensates, correction terms for the activity coefficients may be added to the equations. This is currently under consideration.

Statistical analysis

All statistical analyses were performed in GraphPad Prism 8 (GraphPad Software). Data are presented as means ± SEM from at least 3 independent biological replicates, unless indicated otherwise. The statistical significance was analysed either by 2-tailed Student's t test or one-way ANOVA followed by Bonferroni's multiple-comparison.

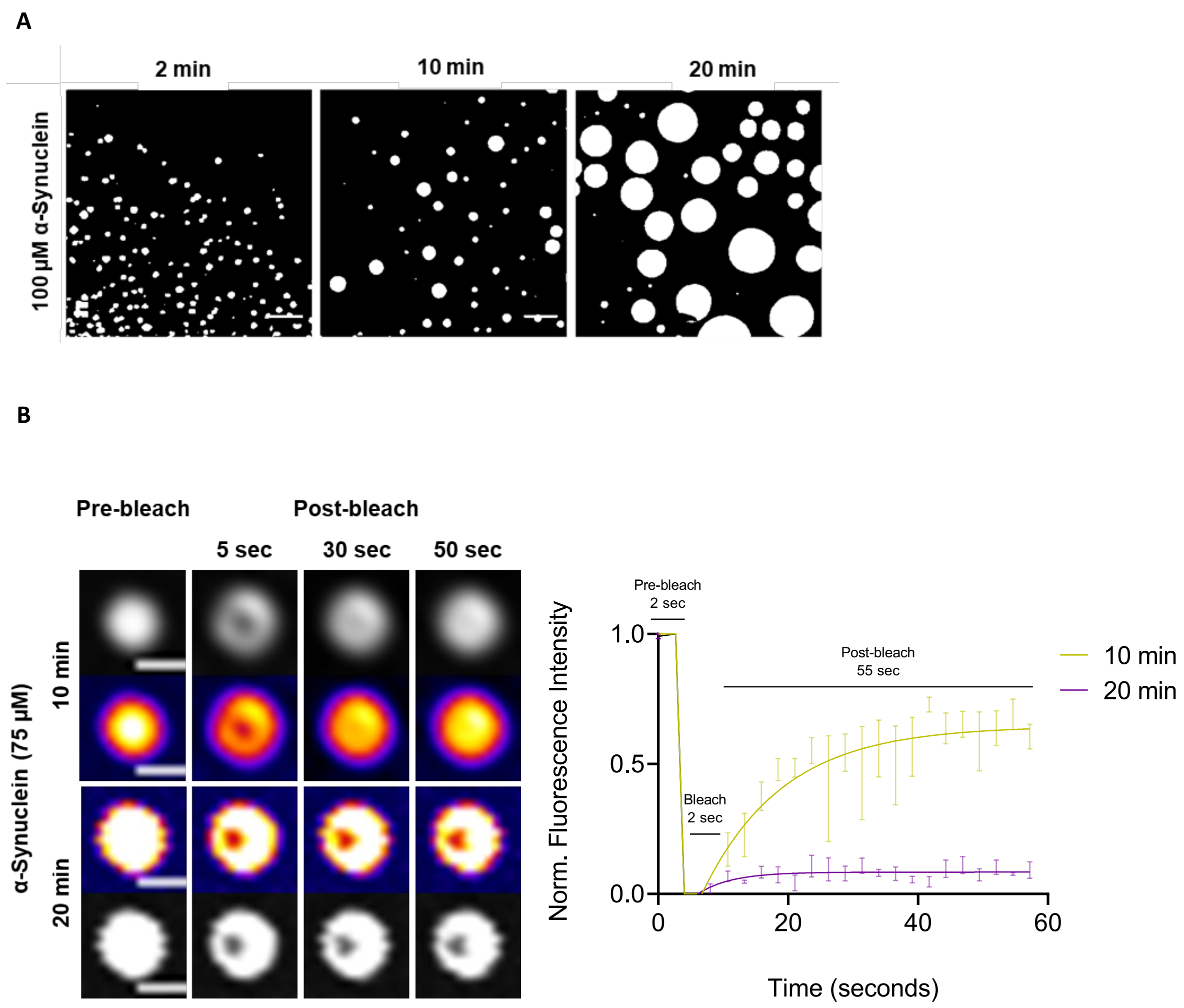

Supplementary Figure 1. α-Synuclein condensates undergo a transition from liquid-like to solid-like forms over time. (**A**) Fluorescence microscopy images displaying formation of α-synuclein condensates (75 µM) at 2, 10 and 20 min. The scale bar indicates 10 µm. (**B**) FRAP measurements of 75 µM α-synuclein condensates at 10 and 20 min quantify the change in dynamics of condensates over time. The images correspond to the region of interest with pre-bleach and post-bleach condensates at 5, 30 and 50 sec for each condition. The scale bar indicates 2 µm. The data represent the mean ± SEM of n=4 individual experiments.

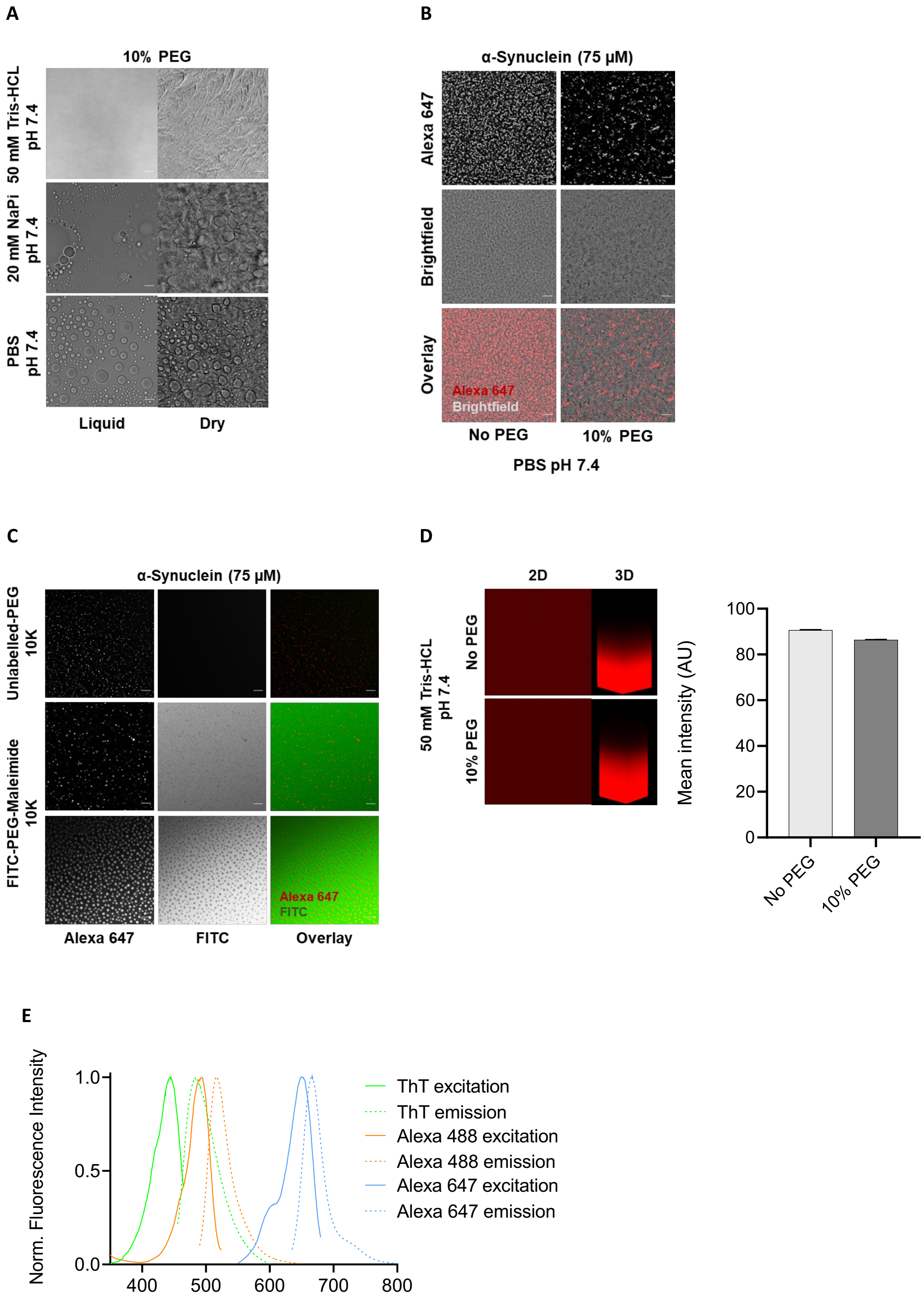

Supplementary Figure 2. Analysis of the use of 10% PEG to study α-synuclein phase separation under various buffer conditions. (A) Bright-field images showing the formation of PEG condensates under various buffer conditions at pH 7.4 in the absence of α-synuclein and with 10% PEG: 50 mM Tris-HCl, 20 mM NaPi, and PBS. PEG condensates were observed when 10% PEG and 20 mM NaPi

or PBS were combined. No PEG condensates were observed when 10% PEG and 50 mm Tris-HCl were used. (**B)** Fluorescence microscopy images showing the observed condensate formation of 75 μM α-synuclein (labelled with Alexa Fluor 647) in PBS in the presence and absence 10% PEG. **(C)** Microscopy images of 75 μM α-synuclein (labelled with Alexa Fluor 647) in 50 mM Tris-HCl with the presence of 10K-unlabelled PEG (10%) and 10K FITC (fluorescein isothiocyanate) labelled-PEG-maleimide (10%). **(D)** Alexa Fluor 647 fluorescence intensity in 50 mM Tris-HCl in the presence and absence of 10% PEG. **(E)** Excitation and emission spectra of ThT, Alexa Fluor 488 and Alexa Fluor 647 in the presence of 50 mM Tris-HCl.

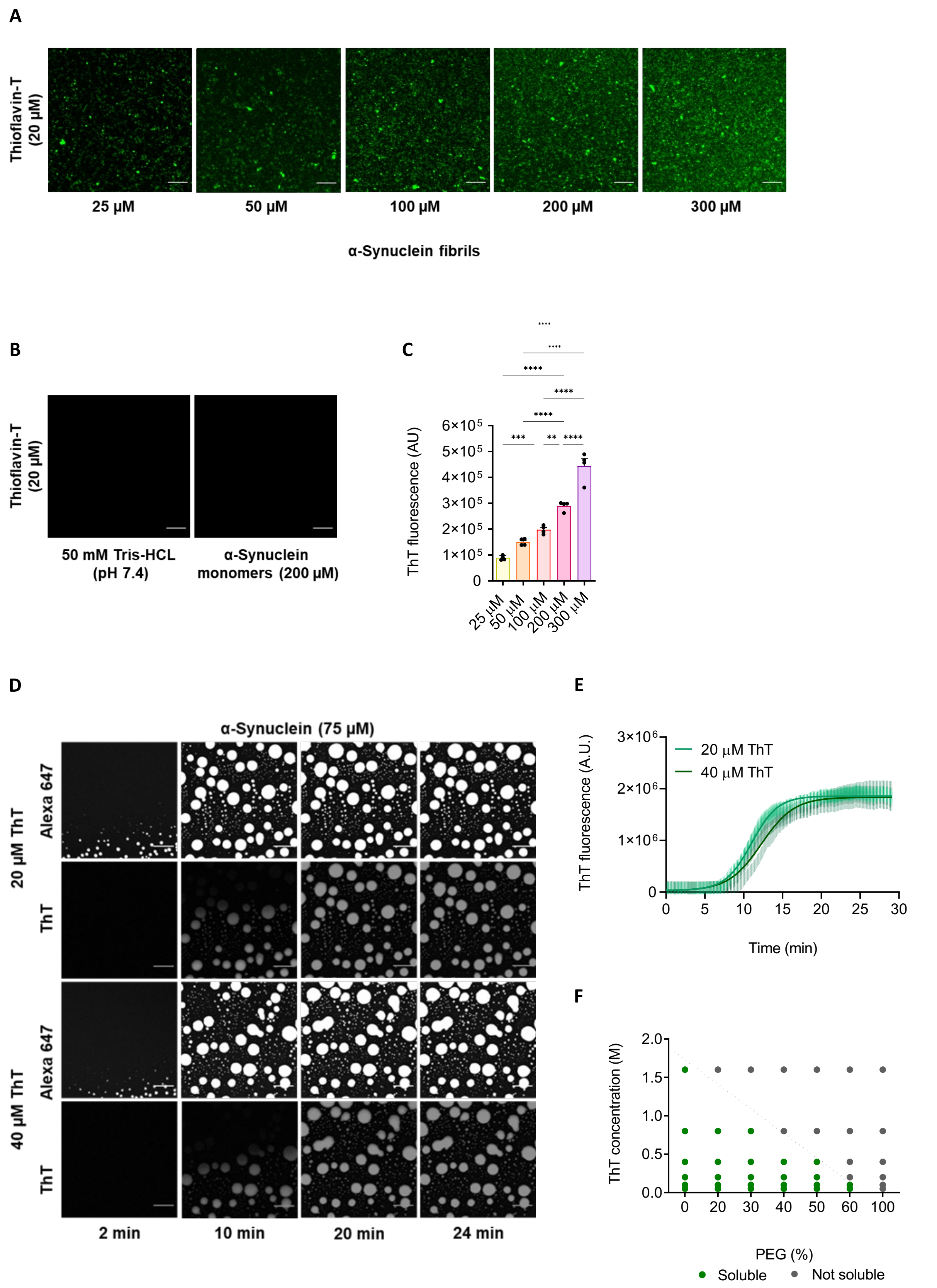

Supplementary Figure 3. Analysis of the use of ThT as a reporter fluorescent dye for α-synuclein fibril formation within liquid-like condensates. (A) Fluorescence microscopy images highlighting the concentration dependence of the ThT fluorescence intensity on the amyloid fibril load with

increasing concentration of α-synuclein preformed fibrils (25, 50, 100, 200 and 300 μM) with 20 μM ThT in 50 mM Tris-HCl at pH 7.4. The scale bar represents 10 μm. **(B)** In the absence of amyloid fibrils, ThT fluorescence is not observed. Monomeric α-synuclein (200 μM) and 50 mM Tris-HCl at pH 7.4 in the presence of 20 μM ThT was used for control experiments. **(C)** Quantification of the ThT (20 μM) fibril-dependent fluorescence intensity of the representative images shown in panel A for 25 μM (yellow), 50 μM (orange), 100 μM (red), 200 μM (magenta) and 300 μM (purple) α-synuclein preformed fibrils. All experiments were performed in 50 mM Tris-HCl (pH 7.4) in the presence of 20 μM ThT. **(D)** Fluorescence imaging of α-synuclein condensate and amyloid formation using 20 and 40 μM ThT. The images represent an area of the sample that was tracked over time, showing no detectable changes after 10 min. **(E)** Quantification of 75 μM α-synuclein ThT fluorescence over time of the images shown in panel D for 20 (light green) and 40 (dark green) μM ThT. ThT was measure every 2 sec over 30 min. **(F)** Phase diagram showing the solubility of ThT (powder) at different concentrations for different PEG concentrations. Grey dots indicate ThT insolubility, and green dots indicate ThT solubility. A dotted black line is used for guidance**.** The data represent the mean ± SEM of n=4 individual experiments. One-way ANOVA., $^{**}P < 0.01$, $^{***}P < 0.001$, $^{****}P < 0.0001$.

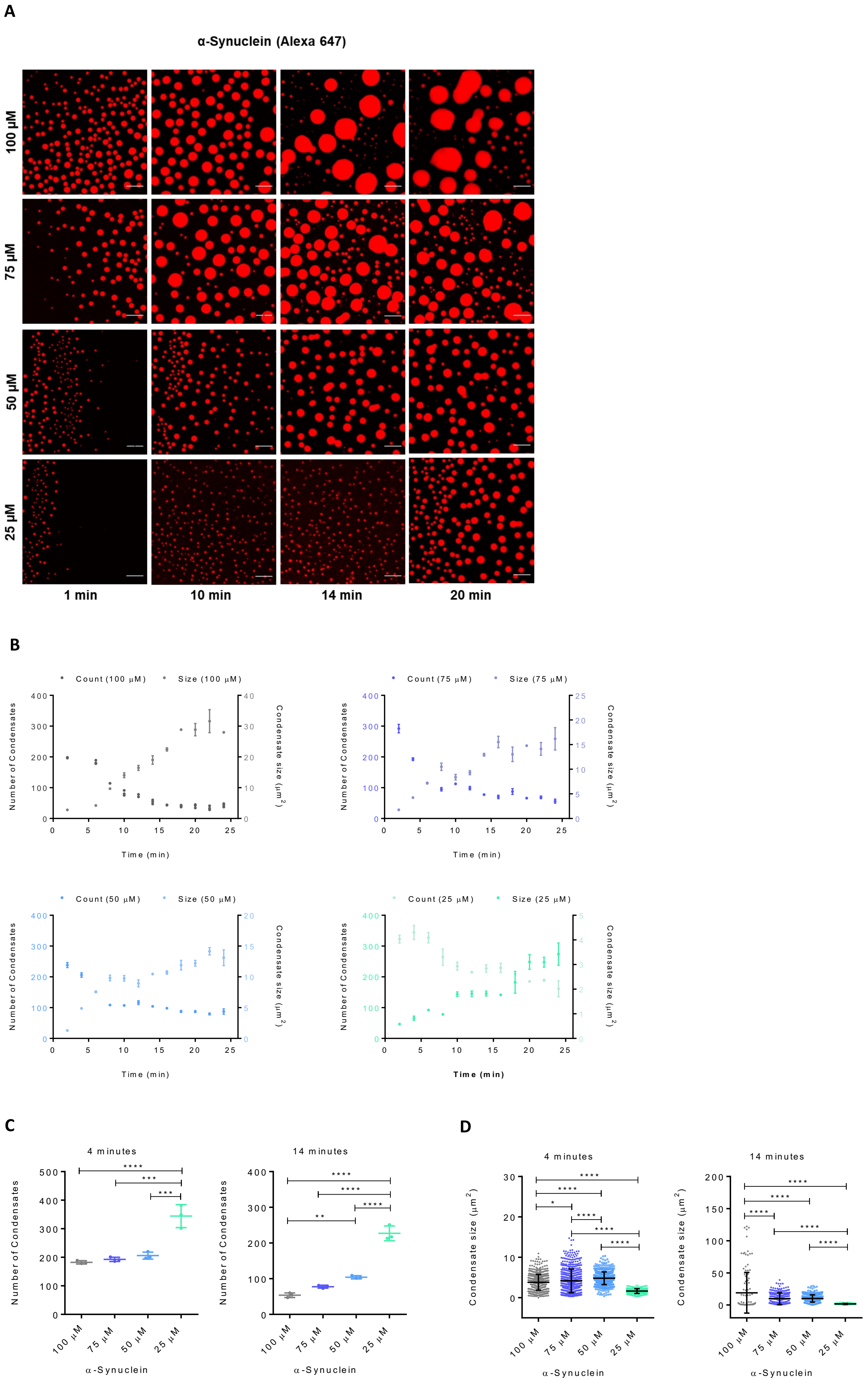
A
α-Synuclein (Alexa 647)
100 µM
75 µM
50 µM
25 µM
1 min
10 min
14 min
20 min
B
Count (100 µM)
Size (100 µM)
Count (75 µM)
Size (75 µM)
Count (50 µM)
Size (50 µM)
Count (25 µM)
Size (25 µM)
Number of Condensates
Condensate size (µm²)
Time (min)
C
4 minutes
14 minutes
Number of Condensates
100 µM
75 µM
50 µM
25 µM
α-Synuclein
D
4 minutes
14 minutes
Condensate size (µm²)
α-Synuclein

Supplementary Figure 4. α-Synuclein forms liquid condensates at physiological concentrations under crowding conditions. (A) Fluorescence microscopy images of α-synuclein forming µm-sized condensates (labelled with Alexa Fluor 647) in the presence of crowding agent (10% PEG) in 50 mM Tris-HCl at physiological concentrations (25 - 100 µM) and pH (7.4) over time. The scale bar represents 10 µm. **(B)** Quantification of condensates number and average size at indicated time points at physiological concentrations of 100 (grey), 75 (blue), 50 (cyan) and 25 (turquoise) µM of α-synuclein under crowded conditions in 50 mM Tris-HCl and pH 7.4. The inverse relation between number (dark colour) and size (light colour) illustrates the growth of the condensates over time. **(C,D)** Quantification of total number (C) and size distribution (D) of condensates at 4 and 14 min after phase separation**.** All experiments were performed in 50 mM Tris-HCl at pH 7.4 in the presence of 10% PEG. The data represent the mean ± SEM of n=3 individual experiments. One-way ANOVA. *$P < 0.05$, **$P < 0.01$, ***$P < 0.001$, ****$P < 0.0001$.

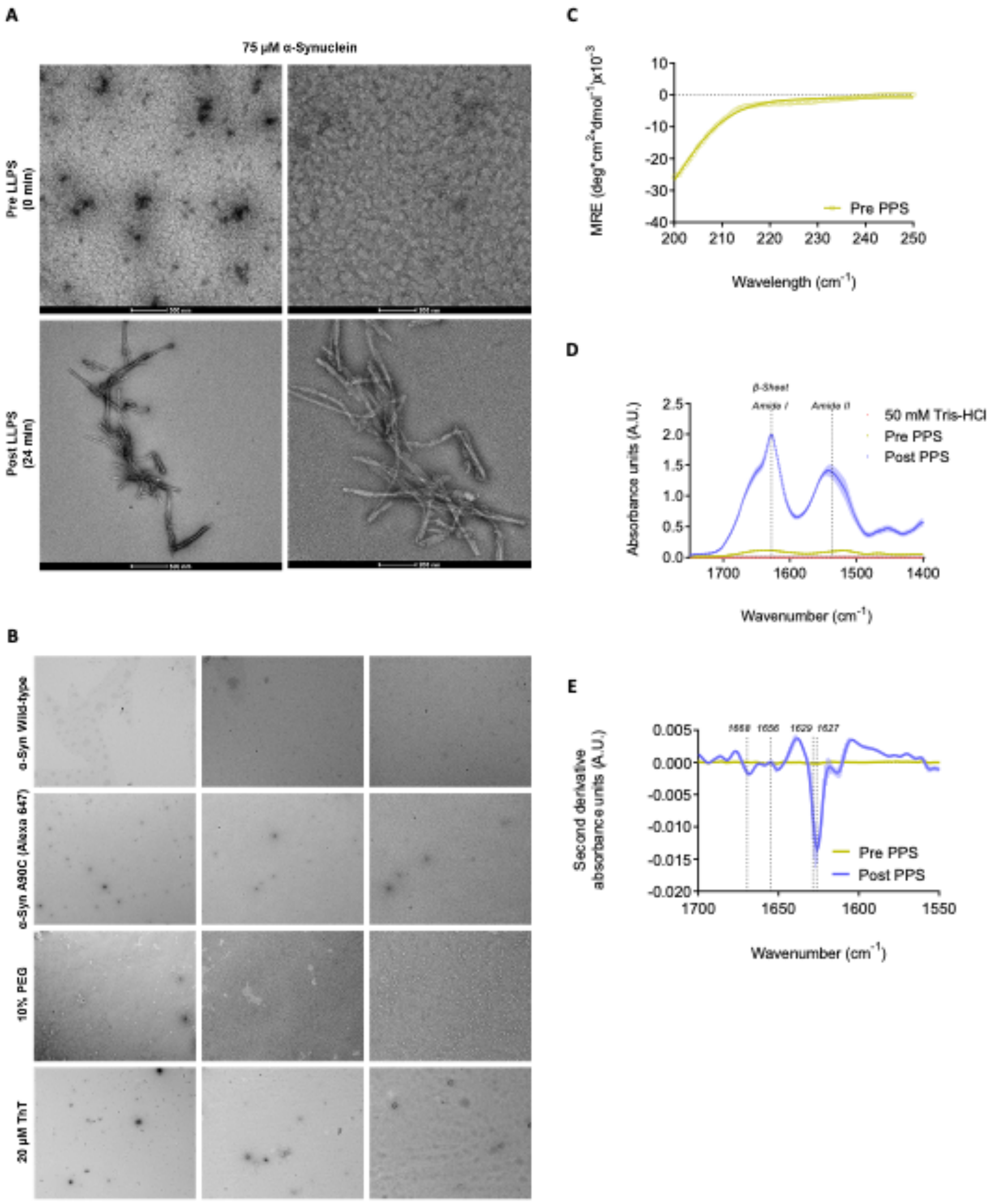

Supplementary Figure 5. α-Synuclein condensates evolve into fibrillar aggregates over time. (A) TEM images highlighting uranyl acetate α-synuclein species before (0 min) and after (24 min) phase separation. Monomeric species are highlighted at 0 min, whilst the formation of amyloid fibrils of α-synuclein post phase separation are validated at 24 min. Experiments were performed with 75 µM α-synuclein in 50 mM Tris-HCl, pH 7.4, 10% PEG and 20 µM ThT. **(B)** TEM images showing the absence of aggregates after 24 min of incubation with each individual component of the α-synuclein ThT aggregation assay through phase separation. 1700x, 5000x and 11500x direct magnification from left

to right panels were taken respectively. (**C)** Circular dichroism (CD) spectra confirming unfolded, random coil conformation of α-synuclein (75 μM) in solution, within the assay prior to phase separation. **(D)** Fourier-transform infrared (FTIR) spectra of 50 mM Tris-HCl (coral), sample pre-phase separation (yellow) and recovered products from phase separation assay (blue) (75 μM α-synuclein) highlighting the amide I and amide II regions. (**E)** Second derivative FTIR spectra of deconvoluted amide I region from panel F showing the band frequency assignments assigned to α-helix (~1656 cm^{-1}), β-sheet (~1627 cm^{-1}) and turn (~1668 cm^{-1}) structures pre (yellow) and post (blue) phase separation of α-synuclein (75 μM). Experiments were performed in 50 mM Tris-HCl at pH 7.4.

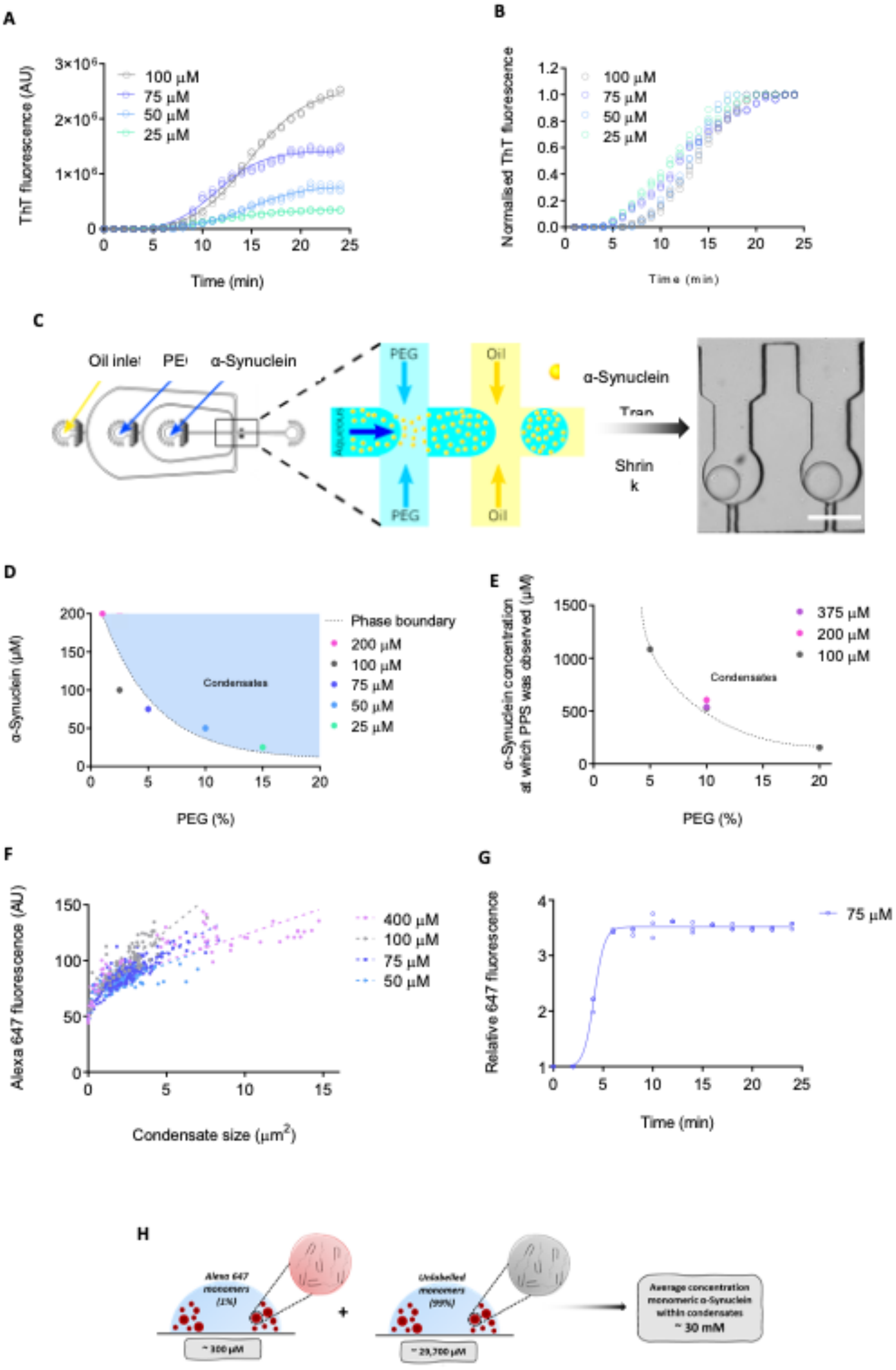

Supplementary Figure 6. The concentration of α-synuclein required for phase separation depends on the type and concentration of the crowding agent used. (A,B) Within condensates, normalised

aggregation data at four α-synuclein concentrations (100 (grey), 75 (blue), 50 (cyan) and 25 (turquoise) μM), yielded almost no variations between different concentrations. **(A)** Quantification of ThT emission over a 24 min period for the four different α-synuclein concentrations. **(B)** Normalised representative aggregation kinetic traces of α-synuclein shown in panel A. All experiments were performed in 50 mM Tris-HCl at pH 7.4 in the presence of 10% PEG and 20 μM ThT. **(C)** Schematic illustration of the microfluidic device used to obtain the concentration at which α-synuclein phase separation is observed. The device is pumped with oil, PEG and α-synuclein: (i) PEG and α-synuclein are mixed first to make droplets, and (ii) the droplets are then pushed and trapped in a chamber enclosed by fluorinated oil, for observation using microscopic techniques. The scale bar represents 100 μm. **(D)** Phase diagram showing the relationship between different α-synuclein concentrations, PEG concentration and the concentration at which phase separation is observed in the ThT-based assay with varying PEG and protein concentrations. An approximate phase boundary is represented by a black line. **(E)** Phase diagram showing the relationship between different α-synuclein concentrations, PEG concentration and the concentration at which phase separation is observed in the microfluidics-based assay with varying PEG and protein concentrations. A dotted black line is used for guidance as the trend follows the same trend shown in panel B. Values are detailed in **Supplementary Table 1.** **(F)** Scatter plot showing the relationship between condensate size and Alexa Fluor 647 fluorescence for individual condensates of 400 (light purple), 100 (grey), 75 (blue) and 50 (cyan) μM α-synuclein, 10 min from the onset of phase separation (n > 70 condensates per concentration); the linear regression coefficient r^2 is 0.63 for 400 μM, 0.71 for 100 μM, 0.70 for 75 μM and 0.44 for 50 μM. **(G)** Progression of Alexa Fluor 647 fluorescence intensity with time for α-synuclein (75 μM), which was measured at > 1 min and normalised against the first time point. After 5 min from the onset of phase separation, the Alexa Fluor 647 fluorescence intensity remains constant. **(H)** Estimated total α-synuclein concentration within condensates, which is approximately 30 mM; the total α-synuclein concentration (labelled and unlabelled) within condensates was estimated at an average of 33.5, 31.9, 28.4 and 25.8 mM for samples at 400, 100, 75 and 50 μM α-synuclein. Data are from a representative experiment that was repeated at least three times with similar results. All phase separation experiments were performed in 50 mM Tris-HCl, pH 7.4 and 10% PEG unless otherwise stated.

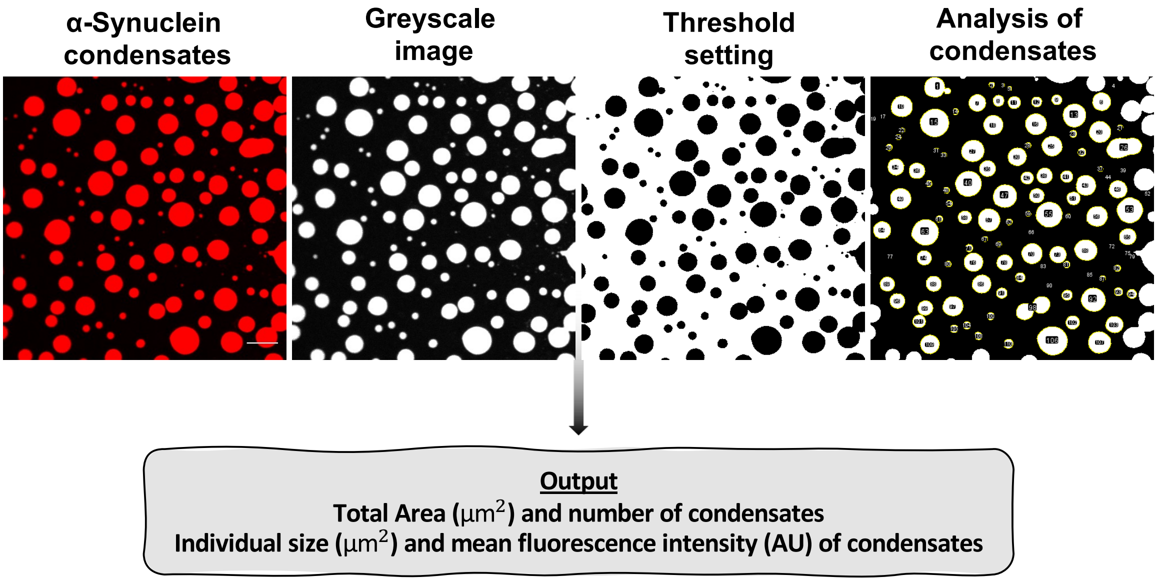

Supplementary Figure 7. Fluorescence image analysis of α-synuclein condensates. Images of wild-type α-synuclein, containing 1 molar % A90C α-synuclein labelled with Alexa Fluor 647 were acquired on a Leica TCS SP8 inverted confocal microscope system. All images within an experiment were acquired using identical confocal settings (scan speed, resolution, magnification, laser intensity, gain, and offset). Images were analysed by applying threshold functions in ImageJ software that identified the phase separated α-synuclein condensates and excluded the background of the image. All condensates within the threshold limits were analysed for total area (μm^2), number and average size of individual condensates (μm^2) and mean fluorescence intensity of individual condensates (arbitrary units). The scale bar represents 10 μm.

Supplementary Table 1. Values of the concentrations of α-synuclein, ThT and PEG at which phase separation was observed in the microfluidics measurements described in this work.

α-Synuclein monomer concentration (µM)	PEG concentration (%)	α-Synuclein concentration at which LLPS was observed (µM)	Standard deviation (STD)	ThT concentration at which LLPS was observed (µM)	Standard deviation (STD)	PEG concentration at which LLPS was observed (%)	Standard deviation (STD)
**100**	**5**	1084	10.8436	218.7182975	4.019814	54.21799085	1.380908
**100**	**10**	527	15.76637	78.62773753	3.153274	39.31386876	1.576637
**100**	**20**	154	4.012245	30.93155071	0.856264	30.85039194	0.802449
**200**	**10**	604	16.85452	65.0901998	6.164437	32.5450999	3.082218
**375**	**10**	538	13.03347	57.39246923	1.390237	28.69623462	0.695118

Movie S1. Formation of α-synuclein condensates (75 µM) at the edge of a drop in 50 mM Tris, pH 7.4 and 10% PEG; the scale bar represents 20 µm.

Movie S2. Fusion events of several α-synuclein condensates after 7 min post the onset of phase separation; the scale bar represents 20 µm,

Movie S3. α-Synuclein condensates formation and maturation, and α-synuclein aggregation at near-physiological concentration (75 µM) and conditions (50 mM Tris, pH 7.4) in the presence of 20 µM ThT; Alexa 647 channel (red) ThT channel (green) and merged channels; the scale bar represents 10 µm.

Movie S4. Time-lapse of formation of α-synuclein condensates in droplets trapped within the microfluidic device.

Movie S5. 3D rendered composition from Z-stacked images showing preformed fibrils (Alexa Fluor 488 - green) co-localised with α-synuclein condensates (Alexa Fluor 647 - red).

Supplementary References

1. G. Fusco *et al.*, Direct observation of the three regions in α-synuclein that determine its membrane-bound behaviour. *Nat. Comm.* **5**, 1-8 (2014).
2. Z. Toprakcioglu, P. K. Challa, A. Levin, T. P. Knowles, Observation of molecular self-assembly events in massively parallel microdroplet arrays. *Lab Chip* **18**, 3303-3309 (2018).
3. Z. Toprakcioglu *et al.*, Multi-scale microporous silica microcapsules from gas-in water-in oil emulsions. *Soft Matter* **16**, 3082-3087 (2020).
4. Z. Toprakcioglu, T. P. Knowles, Sequential storage and release of microdroplets. *Microsyst. Nanoeng.* **7**, 1-10 (2021).
5. C. Weber, T. Michaels, L. Mahadevan, Spatial control of irreversible protein aggregation. *eLife* **8**, e42315 (2019).
6. G. Meisl *et al.*, Molecular mechanisms of protein aggregation from global fitting of kinetic models. *Nat. Protoc.* **11**, 252-272 (2016).
